# Specializations of the mandibular anatomy and dentition of *Segnosaurus galbinensis* (Theropoda: Therizinosauria)

**DOI:** 10.7717/peerj.1885

**Published:** 2016-03-29

**Authors:** Lindsay E. Zanno, Khishigjav Tsogtbaatar, Tsogtbaatar Chinzorig, Terry A. Gates

**Affiliations:** 1 Paleontology Research Lab, North Carolina Museum of Natural Sciences, Raleigh, North Carolina, United States of America; 2 Department of Biological Sciences, North Carolina State University, Raleigh, North Carolina, United States of America; 3 Institute of Paleontology and Geology, Mongolian Academy of Sciences, Ulaanbaatar, Mongolia; 4 Hokkaido University Museum, Hokkaido University, Sapporo, Japan

**Keywords:** Cretaceous, Dinosaur, Theropod, Anatomy, Ecomorphology, Therizinosaur, Dentition, Evolution, Dietary specializations, Herbivory

## Abstract

Definitive therizinosaurid cranial materials are exceptionally rare, represented solely by an isolated braincase and tooth in the North American taxon *Nothronychus mckinleyi*, the remarkably complete skull of the Asian taxon *Erlikosaurus andrewsi*, and the lower hemimandibles of *Segnosaurus galbinensis*. To date, comprehensive descriptions of the former taxa are published; however, the mandibular materials of *S. galbinensis* have remained largely understudied since their initial description in 1979. Here we provide a comprehensive description of the well-preserved hemimandibles and dentition of *S. galbinensis* (MPC-D 100/80), from the Upper Cretaceous Bayanshiree Formation, Gobi Desert, Mongolia. The subrectangular and ventrally displaced caudal hemimandible, extreme ventral deflection of the rostral dentary, and edentulism of the caudal dentary of *S. galbinensis* are currently apomorphic among therizinosaurians. Unique, unreported dental traits including lingually folded mesial carinae, development of a denticulated triangular facet on the distal carinae near the cervix, and extracarinal accessory denticles, suggest a highly specialized feeding strategy in *S. galbinensis*. The presence of triple carinae on the distalmost lateral tooth crowns is also unique, although may represent an abnormality. Contrasted with the simplistic dentition of the contemporaneous therizinosaurid *E. andrewsi*, the dentition of *S. galbinensis* is indicative of niche partitioning in food acquisition, processing, or resources among known therizinosaurids inhabiting Asian ecosystems in the Late Cretaceous. Although not quantitatively correlated with diet, this suite of specializations is otherwise unique among theropod dinosaurs and supports derived inferences of facultative or obligate herbivory in therizinosaurids, ultimately adding novel information to our understanding of ecomorphology in theropods.

## Introduction

Therizinosaurians exhibit a highly unusual bauplan among theropod dinosaurs, a condition that precluded even a rudimentary understanding of their evolutionary relationships for decades (Maleev, 1954; Rozhdestvensky, 1970; Paul, 1984; Gauthier, 1986; Sereno, 1989; Russell & Dong, 1993; Xu, Tang & Wang, 1999). Chief among their anatomical oddities are specializations of the mandible and dentition analogous with those of herbivorous dinosaurs that have since been quantitatively correlated with diet (Zanno et al., 2009; Zanno & Makovicky, 2011). Although newly discovered species have helped to resolve the relationships among early diverging taxa and illuminate initial evolutionary transitions, new fossil discoveries have not improved relationships among specialized, Late Cretaceous therizinosaurids, which continue to be uninformative despite intensifying character and taxon sampling (Kirkland et al., 2005; Zanno, 2010; Pu et al., 2013).

The most serious impediment to reconstructing the phylogenetic history of Therizinosauria is the fact that the majority of therizinosaurids are known from non-overlapping, highly fragmentary remains, and that to date, most therizinosaurid specimens remain inadequately described and photodocumented (Maleev, 1954; Barsbold, 1976; Dong, 1979; Perle, 1979; Barsbold & Perle, 1980; Barsbold, 1983; Dong & Yu, 1997). Some of the most informative materials known for Late Cretaceous therizinosaurids (e.g., rare elements of the cranial skeleton of *Erlikosaurus andrewsi* Barsbold & Perle, 1980 and *Segnosaurus galbinensis* Perle, 1979; the extensive vertebral series of *Nanshiungosaurus brevispinus* and “*Nanshiungosaurus*” *bohlini*) have had the confounding issue of being intermittently inaccessible for further study (Zanno, 2010).

**Figure 1:**
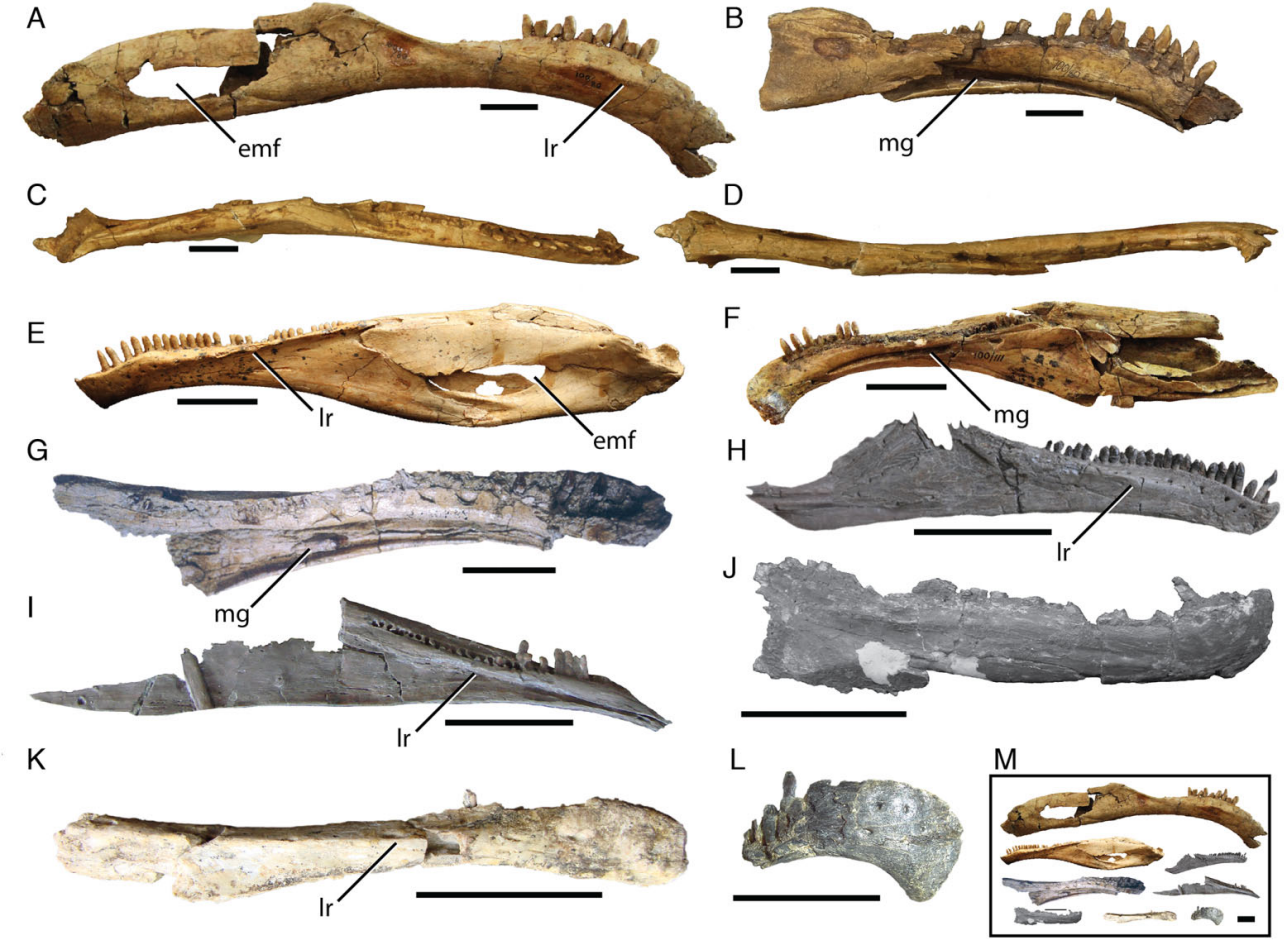
Variation in therizinosaurian mandibular morphology. *Segnosaurus galbinensis* MPC-D 100/80 right hemimandible in (A) lateral, (C) dorsal, and (D) ventral views and (B) left hemimandible in medial view; *Erlikosaurus andrewsi* (MPC-D 100/111) (E) left hemimandible in lateral view and (F) right hemimandible in medial view (D); (G) *Alxasaurus elesitaiensis* (IVPP 88402) left dentary in medial view; (H) *Jianchangosaurus yixianensis* (41HIII-0308A) right dentary in lateral view; reproduced and modified from Pu et al. (2013). (I) *Beipiaosaurus inexpectus* (IVPP V11559) left dentary in medial view; (J) *Falcarius utahensis* (UMNH VP 14529) right dentary in lateral view; (K) *Eshanosaurus deguchiianus* (IVPP V11579) right dentary in lateral view (L) *Neimongosaurus yangi* (LH V0001) right rostral dentary in lateral view; (M) shown to same scale. Abbreviations: emf, external mandibular fenestra; lr, labial ridge; mg, Meckelian groove. Scale bar equals 3 cm.

Despite the dearth of cranial skeletal materials among therizinosaurians generally, elements of the lower mandible are among the most widely, if still rarely represented (Fig. 1). A series of recent papers provides a thorough description of the skull of *E. andrewsi* (Lautenschlager et al., 2012; Lautenschlager et al., 2014), building on the substantial description of Clark, Perle & Norell (1994). However, the nearly complete hemimandibles of *S. galbinensis* (Perle, 1979; Barsbold & Perle, 1980) have not yet been adequately described or figured. Moreover, descriptions of teeth in specialized therizinosaurians are exceedingly rare. Brief descriptions of the dentition of *Neimongosaurus yangi*, *E. andrewsi*, *S. galbinensis*, and a single isolated, poorly preserved tooth associated with *Nothronychus mckinleyi* are published (Kirkland & Wolfe, 2001; Clark, Maryánska & Barsbold, 2004; Hedrick et al., 2015); however, detailed study and adequate figures are lacking for all but *E. andrewsi* (Clark, Perle & Norell, 1994; Lautenschlager et al., 2014) and *No. mckinleyi* (Hedrick et al., 2015).

Here we present a detailed description of the unique morphology of the lower mandible and dentition of the *S. galbinensis* (MPC-D 100/80), from the Upper Cretaceous Bayanshiree Formation, Gobi Desert, Mongolia. We document unexpectedly complex dental traits in this taxon and illustrate abundant and previously unrecognized mandibular anatomy of phylogenetic utility, placing special emphasis on comparisons with the coeval Bayanshiree therizinosaurid *E. andrewsi*.

## Materials and Methods

We describe and figure the lower mandibular anatomy of MPC-D 100/80 from the Upper Cretaceous Bayanshiree Formation, Gobi Desert, Mongolia using a Canon EOS 5D Mark II 21.1 megapixel full-frame CMOS digital single-lens reflex camera for image capture. Tooth images were captured with a Dinolite Edge AM4815ZT polarizing, extended depth of field microscope. Tooth terminology follows Smith & Dodson (2003) and Hendrickx, Mateus & Araújo (2015). **MPC-D**: Institute of Paleontology and Geology (Mongolian Paleontological Center), (Mongolian Academy of Sciences, Ulaanbaatar, Mongolia, formerly IGM).

## Results

**Figure 2:**
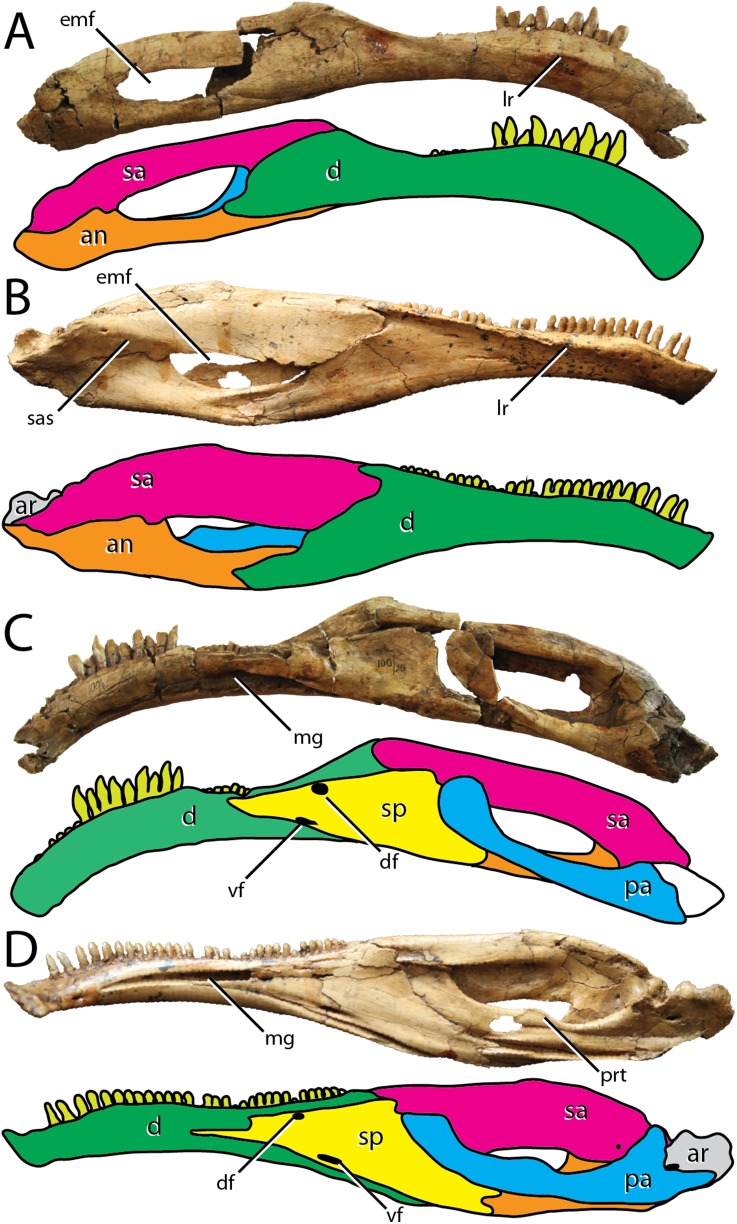
Comparisons between the hemimandibles of the Bayanshiree therizinosaurians *Segnosaurus galbinensis* and *Erlikosaurus andrewsi*. Right hemimandible of *Segnosaurus galbinensis* in (A) lateral view and (C) medial views; left hemimandible of *Erlikosaurus andrewsi* in (B) lateral view (reversed) and (D) medial view (reversed). Dentary (green), surangular (pink), angular (orange), splenial (yellow), prearticular (blue), articular (gray), teeth (light green). Prearticular reconstructed in drawing of (C). Abbreviations: an, angular; ar, articular; d, dentary; df, dorsal foramen splenial; emf, external mandibular fenestra; lr, labial ridge; mg, Meckelian groove; pr, prearticular; prt, prearticular tab; sa, surangular; sp, splenial; sas, surangular shelf; vf, ventral foramen splenial. Elements not to scale.

**Figure 3:**
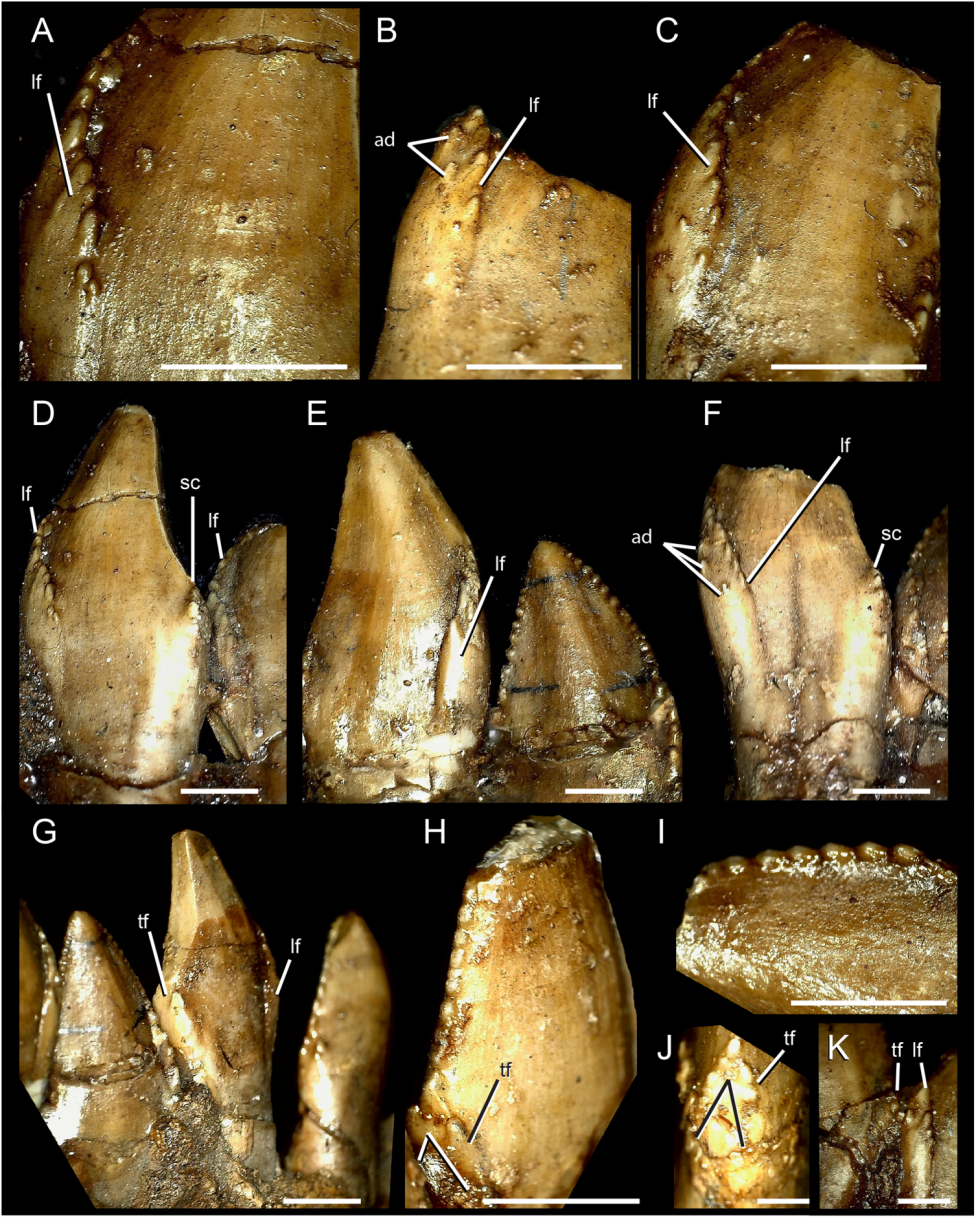
Dentary teeth of *Segnosaurus galbinensis* MPC-D 100/80. (A–H) mesial dentary teeth in lingual view; (I) mesial denticles in lingual view; (J) magnification of triangular prong (bifurcated distal carina); (K) magnification of junction between folded mesial carina and triangular facet near base of crown on distal carina. (A) crown 8; (B) crown 13; (D) crowns 8 and 9 from left to right; (E) crowns 5 and 4 from left to right; (F) crown 6; (G) crowns 5 through 2 from left to right; (I) crown 6. Abbreviations: lf, lingual folding of the mesial carina; ad, accessory (extracarinal) denticles; tf, triangular facet. Scale bar 3 mm (A–I) and 1 mm (J, K).

Two hemimandibles are preserved among the holotype materials of *S. galbinensis* (MPC-D 100/80) (Figs. 1A and 1B). The right hemimandible (Fig. 1A) is nearly complete, missing only the caudalmost aspect (including a portion of the articular, prearticular, and angular) and the rostrodorsal portion of the mandibular symphysis. It is approximately 379 mm in length (Table 1). The left hemimandible (Fig. 1B) is fragmented, preserving a nearly complete dentary and splenial, as well as the rostralmost portions of the surangular and angular. Crushing has displaced the rostralmost surangular ventrally and medially on the right hemimandible (Figs. 2A and 3A) adding to its unusual shape as preserved. In medial view, the rostral aspect of the prearticular on this element has been displaced caudodorsally (Fig. 2C). Relatively little distortion is evident on the left hemimandible, other than slight dorsal displacement of the splenial. In general, the mandibular elements of *S. galbinensis* are relatively robust and amorphous compared to the more gracile, superficially detailed mandible of *E. andrewsi* (Figs. 1A–1D). The majority of tooth crowns have been damaged since collection, and most are missing apices.

**Table 1:** Measurements of the right hemimandible of *Segnosaurus galbinensis* (MPC-D 100/80) in mm.

Mandibular rostrocaudal length	379
Minimum dorsoventral height	24.56
Maximum dorsoventral height	55.5
Maximum length of endentulous rostral dentary (rostralmost tooth to center of symphysis)	25.5
Length of tooth row (left)	138.66
Length of tooth row (right)	150.31

### Dentary

In contrast to the relatively simple morphology of early diverging therizinosaurians such as *Falcarius utahensis* (Zanno, 2010), the dentary of *S. galbinensis* is complex in shape. In lateral view, the tooth-bearing aspect is subrectangular and pendant, exhibiting a pronounced rostroventral arc throughout its length that is more extreme than is currently known for any other therizinosaurian (Fig. 2A). When articulated, the hemimandibles form a broadly U-shaped, edentulous mandibular symphysis that projects rostrodorsally as in *E. andrewsi* and *N. yangi* (Zhang et al., 2001). The edentulous region is extensive, spanning 25.5 mm on the right hemimandible. The proportion of the edentulous rostral dentary to the length of the tooth row is approximately 20%, whereas the edentulous region of the dentary is more reduced in *E. andrewsi* (approx. 12% of the length of the tooth row), near absent in *Jianchangosaurus yixianensis* (Fig. 1F), and cannot be calculated for *Beipiaosaurus inexpectus*, and *N. yangi* (Figs. 1G and 1H).

The dorsoventral height of the dentary constricts as it approaches the caudalmost extent of the tooth row (minimum height 24.56 mm), then fans out sharply to meet the surangular in a steeply inclined articulation (Fig. 2A). This morphology contrasts with the gradually expanded caudal aspect of the dentary of *E. andrewsi* (Perle, 1981), which approaches the surangular contact by means of a gentle arc (Fig. 2B). Nearly the entire dentary is dentigerous in *E. andrewsi*, which bears 31 alveoli. In *S. galbinensis* and *J. yixianensis* (Pu et al., 2013) the teeth are restricted to the rostral two-thirds of the dentary and the alveolar count is lower, 24 and 25–28 respectively. A similarly restricted tooth row likely characterizes *B. inexpectus* (Xu, Tang & Wang, 1999) (Fig. 1H). Damage to the caudal aspect of the dentary in *F. utahensis* (28 tooth positions, Kirkland et al., 2005) and *Alxasaurus elestaiensis* (40 tooth positions, Russell & Dong, 1993), prevents a confident assessment of this feature in these species (Figs. 1E and 1I).

As in all therizinosaurians more derived than *F. utahensis*, the dentary of *S. galbinensis* exhibits an inset tooth row demarcated by a labial (lateral) shelf. However, in *S. galbinensis* this shelf is restricted to the caudal aspect of the dentary, and the raised rim defining it is poorly expressed compared to other taxa (Fig. 1A). Specifically, in *S. galbinensis*, a low ridge rises from the lateral surface between the fifth and fourteenth tooth positions to divide the dentary into subequal dorsal and ventral aspects. Caudal to this point, the ridge trends caudodorsally, becoming confluent with the dorsolateral margin of the dentary and forming a faintly depressed, transverse labial shelf. The lateral ridge of *E. andrewsi*, *J. yixianensis*, *B. inexpectus*, is sharper and more pronounced; it is also more dorsally located in these taxa, creating a more extensive and flattened labial shelf that defines the majority of the dentary (Figs. 1C, 1F and 1H).

A row of foramina pierce the dentary just dorsal to the lateral ridge as in *J. yixianensis* and *A. elesitaiensis*, becoming more irregular in the symphyseal region. By contrast, this row of foramina is directly in line with and ventral to the lateral ridge on *E. andrewsi* (Fig. 1C). Subtriangular interdental plates fuse to one another, forming a distinctly crenulate margin in medial view. The Meckelian groove is more ventrally positioned than that of *E. andrewsi*, and maintains a consistent dorsoventral height until approximately the thirteenth tooth position, after which it widens to contact the splenial.

In lateral view, the dentary tapers to a single caudal process that contributes to the rostroventral margin of the external mandibular fenestra and contacts the angular in a simple articulation (Fig. 2A). Although a dorsal process is absent, a small dorsal prong extends from the dentary to be received in a forked articulation with the surangular. This prong is visible in lateral view on *E. andrewsi*; however, in *S. galbinensis* the prong is entirely restricted to the dorsal aspect (Figs. 2A and 2B). The remainder of the external mandibular fenestra is formed by the surangular and angular (Fig. 2A).

### Splenial

The splenial is mediolaterally thickened and subtriangular in lateral view (Fig. 2C). The caudal margin forms a near ninety-degree angle and is grooved to accept the rostroventral aspect of the prearticular in a reinforced articulation. The caudoventral process is shorter than in *E. andrewsi* (Fig. 2D). However, the caudalmost aspect of this process is damaged and it’s not clear how extensive this process was or if the contact with the articular was bifurcated as in *E. andrewsi*. As in *E. andrewsi*, two foramina pierce the rostralmost aspect of the splenial. The ventral foramen is slit-like in outline and located rostral to the subcircular dorsal foramen in *S. galbinensis* (Fig. 2C). *E. andrewsi* exhibits the opposite pattern, in which the dorsal foramen is rostral to the ventral foramen (Fig. 2D).

### Surangular

The main body of the surangular is elongate and dorsoventrally reduced (Fig. 2A) compared to the deep surangular body of *E. andrewsi* and *J. yixianensis* (Figs. 1F and 2B), bearing lateral and medial flanges (surangular bar) of more similar dorsoventral depth. The relatively compressed dorsoventral height of the lateral surangular flange results in a “U”-shaped cross-section in *S. galbinensis* as opposed to the inverted “J”-shaped cross section of other therizinosaurians. A dorsoventrally shallow surangular also contributes to an expanded external mandibular fenestra in *S. galbinensis* when compared to that of *E. andrewsi*.

The main body of the surangular of *S. galbinensis* projects subhorizontally from the glenoid region to meet the caudal aspect of the dentary at an obtuse angle (Fig. 2A). This contrasts strongly with the gently arched dorsal margin of the surangular in *E. andrewsi*, which is subparallel with the dorsal outline of the dentary (Fig. 2B). The lateral aspect of the surangular is flat, lacking the pronounced, subhorizontally trending shelf bridging the caudodorsal external mandibular fenestra and articular in *E. andrewsi*. Medially, the surangular bar originates just dorsal to the caudal margin of the external mandibular fenestra, and extends rostrally to contact the dentary. It is robust and rounded throughout its full length, rather than tapering to a sharp ridge rostrally as in *F. utahensis* (Zanno, 2010) and *E. andrewsi* (Lautenschlager et al., 2014).

### Angular

The angular of *S. galbinensis* is rostrocaudally extensive and dorsoventrally shallow, mirroring the structure of the surangular (Fig. 2A). The main body is straightened and the ventral margin is flat. Together the caudolateral elements of the hemimandible combine to create an elongate, subrectangular outline that is otherwise distinct among therizinosaurians. As preserved, the rostral aspect of the angular of *S. galbinensis* is comprised of a single, elongate rostroventral process that tapers to a splint in lateral view (Fig. 2A) and buttresses the caudoventral aspect of the dentary in a grooved articulation. In contrast, the angular is rostrally bifurcated in *E. andrewsi*, bracing the caudoventral process of the dentary by means of a ventral and dorsal buttress (Fig. 2B). However, this area on *S. galbinensis* is damaged and therefore, it is likely that the ventral process of the angular of *S. galbinensis* was bifurcated as in *E. andrewsi*. Medially, a ventral shelf projects from the angular of *S. galbinensis* to contact the prearticular. Together they form a transversely wide sulcus along the floor of the mandibular fossa.

### Prearticular

The finger-like prearticular of *S. galbinensis* contacts the surangular and articular caudally, the articular laterally, and the splenial and surangular rostrally (Fig. 2C). The prominent dorsal flange present on *E. andrewsi* is absent in *S. galbinensis*. Consistent with the morphology of the surangular and angular in this taxon, the axis of the prearticular is straight, lacking the curvature observed on *E. andrewsi*. Lowering of the jaw joint in *S. galbinensis*, displaced the caudal prearticular ventrally, where it contributes to the ventral margin of the hemimandible. From this ventrally displaced origination, the prearticular rises obliquely, reaching the rostrodorsal corner of the mandibular fossa. As a result of this orientation and the lack of a dorsal tab, only a small sliver of the caudodorsal margin of the prearticular of *S. galbinensis* is exposed through the external mandibular fenestra when viewed laterally (Fig. 2A). The condition contrasts with that of *E. andrewsi*, wherein the external mandibular fenestra is nearly walled off by extensive exposure of the prearticular in lateral view (Fig. 2B). The main prearticular body of *S. galbinensis* tapers rostrally before terminating as a flattened tab that rests tightly in the angular margin of the caudal splenial (Fig. 2C).

### Dentition

**Figure 4:**
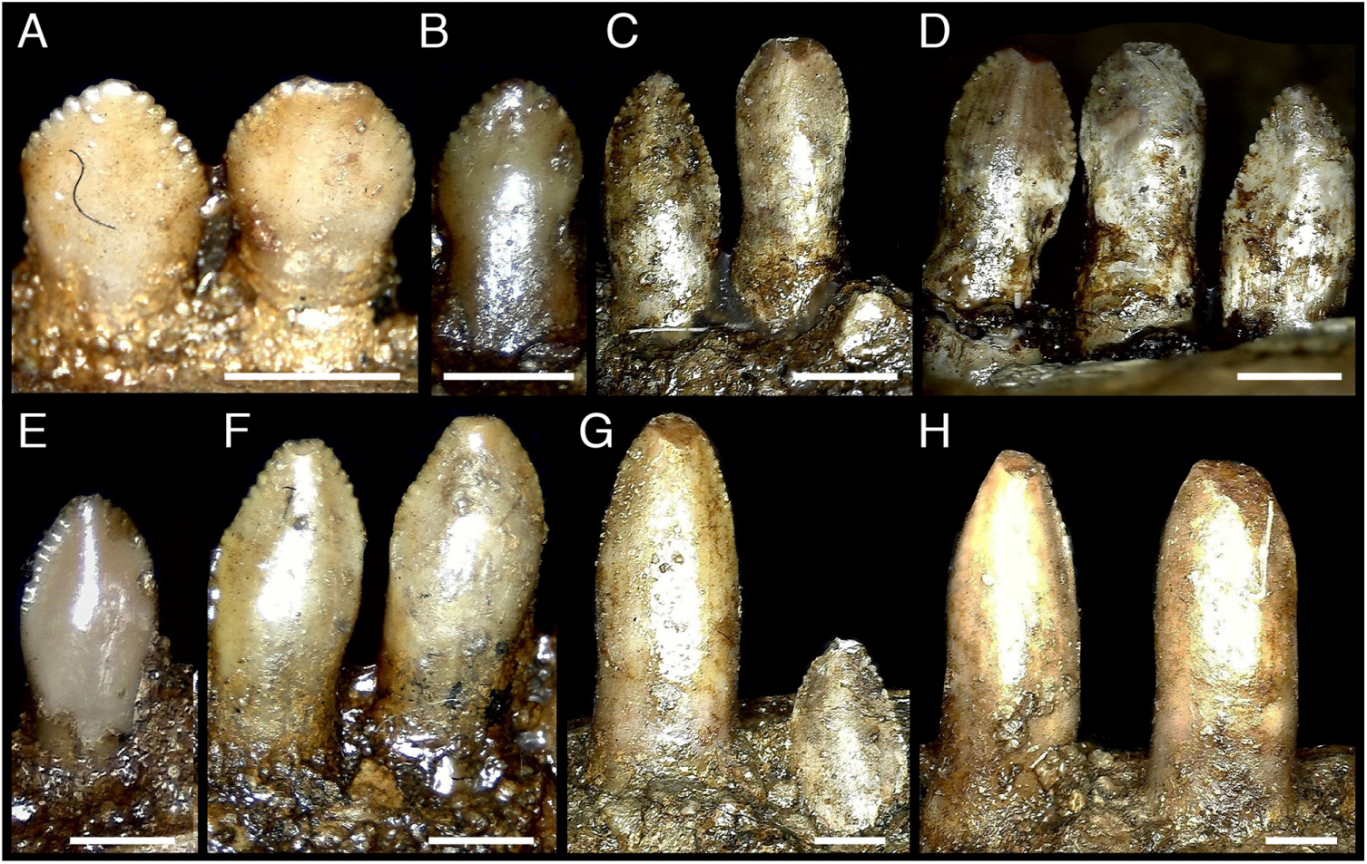
Dentary teeth of *Erlikosaurus andrewsi* (MPC-D 100/111). (A, B) distalmost dentary teeth, (D–F) more mesial dentary teeth; (G) crowns three and four (counting from midline); (H) crowns one and two. All teeth shown in lingual view. Scale bar 1 mm.

**Figure 5:**
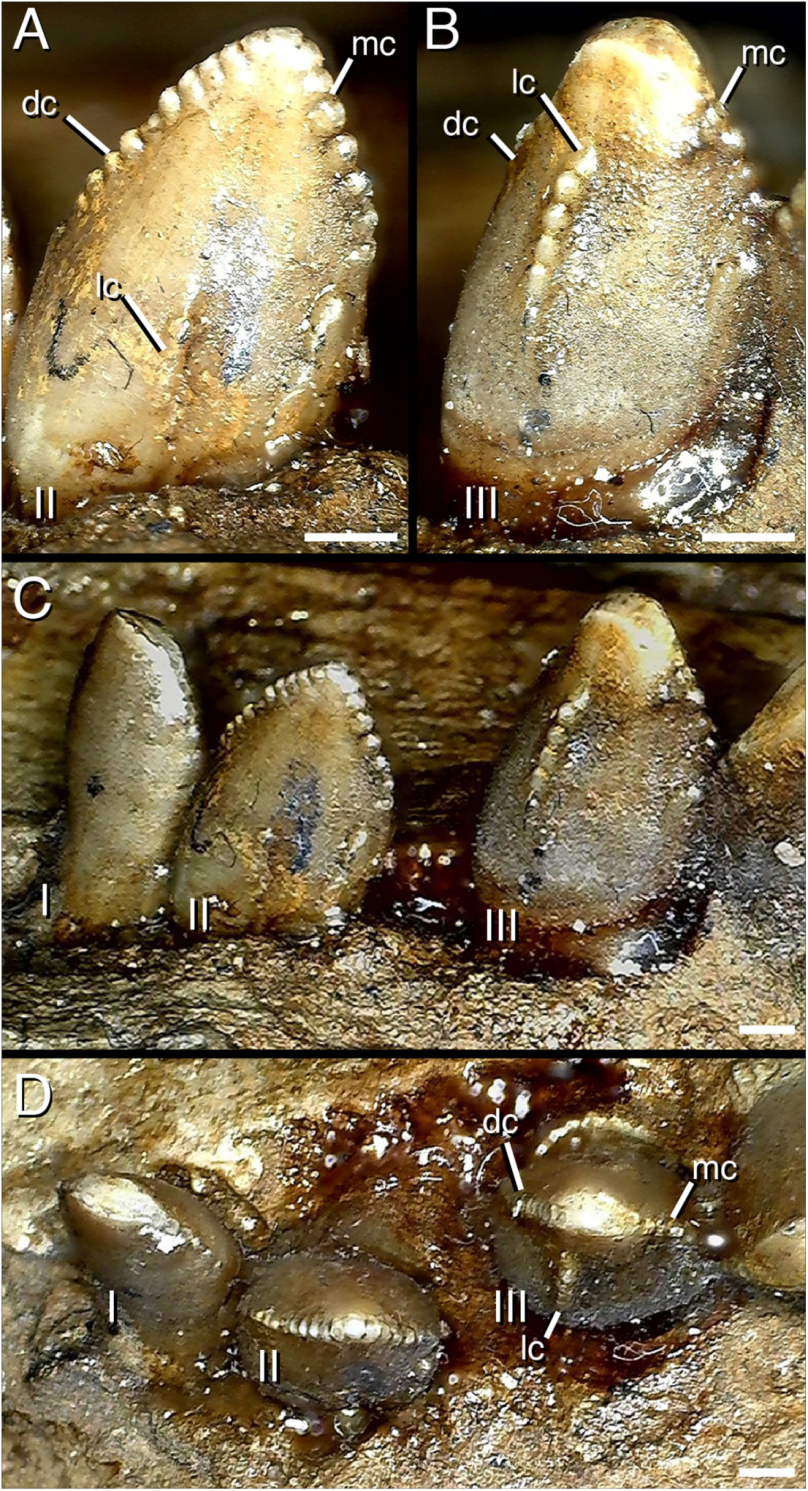
Distalmost dentary teeth (22nd–24th) of *Segnosaurus galbinensis* MPC-D 100/80. (A) 23rd tooth crown in lingual view; (B) 22nd tooth crown in lingual view; (C) 22nd–24th tooth crowns in lingual view; (D) 22nd–24th tooth crowns in occlusal view. Abbreviations: I, 24th tooth; II, 23rd tooth; III, 22nd tooth; dc, distal carina; mc, mesial carina; lc, lingual (accessory) carina. Scale bar 1 mm.

With 24 alveoli in the dentary, *S. galbinensis* exhibits the lowest dentary tooth count, and some of the largest tooth sizes yet known among therizinosaurians. Dentary teeth are folidont (sensu Hendrickx, Mateus & Araújo, 2015), bearing relatively tall, labiolingually compressed, enlarged crowns with a slight apical recurvature along the distal margin. This contrasts with the more diminutive, symmetrical, and simplistic teeth of *E. andrewsi* (Fig. 4). The Crown Base Ratio of *S. galbinensis* increases slightly across the tooth row (from 0.81–0.91; Table 2) reflecting a decrease in labiolingual compression in lateral crowns. Crowns are convex labially and concave lingually as noted by Perle (1979). A thickened longitudinal ridge is present on the lingual aspect near the apical half of the crown and is flanked by weak mesial and distal longitudinal grooves (sensu Hendrickx, Mateus & Araújo, 2015), which extends almost to the level of the cervix (Fig. 3G). A slight lingual depression is present on the lateral teeth and extends apically to the mid-crown height (Fig. 3F). There is no evidence of basal striations, enamel undulations, transverse undulations, marginal undulations, or flutes on the preserved crowns (sensu Hendrickx, Mateus & Araújo, 2015). In general, the mesial 18 teeth (D1–D18) are relatively homodont; however the second tooth crown (D2) possesses a relatively shorter more tapered crown (Crown Height Ratio [CHR] decreases by ∼20% from 2.7–2.14; Table 2) (Fig. 1B), and D1 was likely more tapered as well, although this tooth is not preserved in either dentary. Lateral crowns also decrease in relative height from a CHR of 2.7 on D9 to 2.52 on D18 (Table 2). In *E. andrewsi*, the mesial four to five teeth are conidont (Fig. 4H) and the transition to folidont crowns is gradual (Fig. 4). Most tooth crowns distal to D18 are damaged; however D22–D23 are significantly reduced in size, subconidont, and bear an accessory denticulated, lingual carina (Fig. 5). This accessory lingual carina appears fully denticulated in tooth D23, whereas denticles are restricted to the basal aspect of the crown on D22. To date, triple carinae are otherwise unknown in Theropoda.

**Table 2:** Measurements of the third, ninth, and eighteenth tooth crowns of *Segnosaurus galbinensis* (MPC-D 100/80) in mm.

	III	IX	XVIII
CBL Crown Basal Length, mesiodistal	7.05	6.59	3.99
CBW Crown Basal Width, labiolingual	5.68	5.36	3.64
CH Crown Height	15.07	17.81	10.06
AL Apical Length	16.22	16.24	9.58
CBR Crown Base Ratio (CBW/CBL)	0.81	0.81	0.91
CHR crown height ratio (CH/CBL)	2.14	2.70	2.52

The dentary teeth of *S. galbinensis* are tightly packed, yet not appressed. Tooth crowns approach each other at the point of maximum Mid-Crown Length (MCL sensu Hendrickx, Mateus & Araújo, 2015), and several potentially apomorphic dental features define this region of the mesial-distal crown to crown contact that, to date, have gone unreported. The mesial carinae fold distally to overlap the lingual surface of the tooth (Figs. 3A–3F) on crowns D3–D18. Such a folded carina is definitively absent on the second tooth crown (D2) and likely the first (D1). A series of accessory, extracarinal denticles project from the mesial surface of the carinal fold, roughening the mesial aspect of the crown more broadly (Figs. 3B and 3C).

The distal carinae are also highly modified, bifurcating near the cervix to circumscribe a flattened triangular facet (Figs. 3G, 3H, 3J and 3K). This raised facet projects from the crown surface to contact or closely approach the distally folded mesial carina of the adjacent tooth crown (Fig. 3K) and is present in at least teeth D2–D12. Split carinae are present on other tetanurans and are regarded as an abnormality resulting from trauma, aberrant tooth replacement, or genetic factors (Erickson, 1995). The condition in *S. galbinensisis*, although grossly similar in that the carina bifurcates near the tooth cervix, is uniformly expressed across the lateral teeth of both right and left dentaries, is morphologically distinct in its raised form, and is not an abnormality. Rather, the feature serves to roughen the area between tooth bases.

Waves of replacement teeth encompassing two to three crowns are evident on *S. galbinensis*, as are erupting tooth crowns. In contrast to the majority of other therizinosaurians, wear of distal carinae is observed on some of the fully erupted lateral teeth (e.g., D3, D9) (Clark, Maryánska & Barsbold, 2004). The majority of crown apices are damaged at present; thus, it is difficult to determine the extent of this feature across the tooth row.

The denticles on the mesial and distal carinae are large and bulbous (Figs. 3D and 3E) (approx. 5–6 denticles per 3 mm on the mesial and distal carinae). They are roughly perpendicular to the crown apically, yet parallel to the crown height on the mesial fold and triangular facet on the distal carinae. Toward the apex, they diminish slightly in size. The majority of denticles do not appear to possess caudae or interdenticular sulci, although we note that most of the detail on the tooth crowns including the interdenticular spaces are obscured by a thick consolidant coating. However, denticles on the mesial carina in the region of the carinal fold exhibit interdenticular depressions, adding to the complexity of this particular region on the tooth crown. Enamel texture appears broadly irregular (sensu Hendrickx, Mateus & Araújo, 2015). Roots are subcircular in form.

The 14th alveolus of the right dentary is walled over dorsally by what appears to be pathological bone growth, dividing the tooth row into mesial and distal components. However, corollary pathology of teeth in this region cannot be assessed because all of the crowns distal to this pathology are damaged. On the contralateral dentary, tooth crowns in this region possess triple carinae (as described previously). However, the left dentary does not possess any external indication of pathology that might have prompted this unusual condition. As a result, we have no evidence that this unusual feature is the result of pathology; although such a consideration cannot be ruled out.

## Discussion

### Taxonomic implications

It is difficult to justify adding the unique mandibular and dental traits of *S. galbinensis* to the diagnosis of this taxon given the dearth of comparative materials in other therizinosaurids (e.g., *No. mckinleyi* and *No, graffami*, *Therizinosaurus cheloniformis*, *Na. brevispinus* and *Na. bohlini*, *Enigmosaurus mongoliensis*, *Suzhousaurus megatherium*). Moreover, comparisons between relatively complete postdentary elements can only be made between two therizinosaurians (*S. galbinensis* and *E. andrewsi*); therefore, for many features in which *E. andrewsi* and *S. galbinensis* differ it is currently impossible to determine which character states (if any) are apomorphic relative to the condition of other therizinosaurians. We note that following features add novel character information that may be of phylogenetic utility to evolutionary studies of therizinosaurians and theropods more generally.

**Figure 6:**
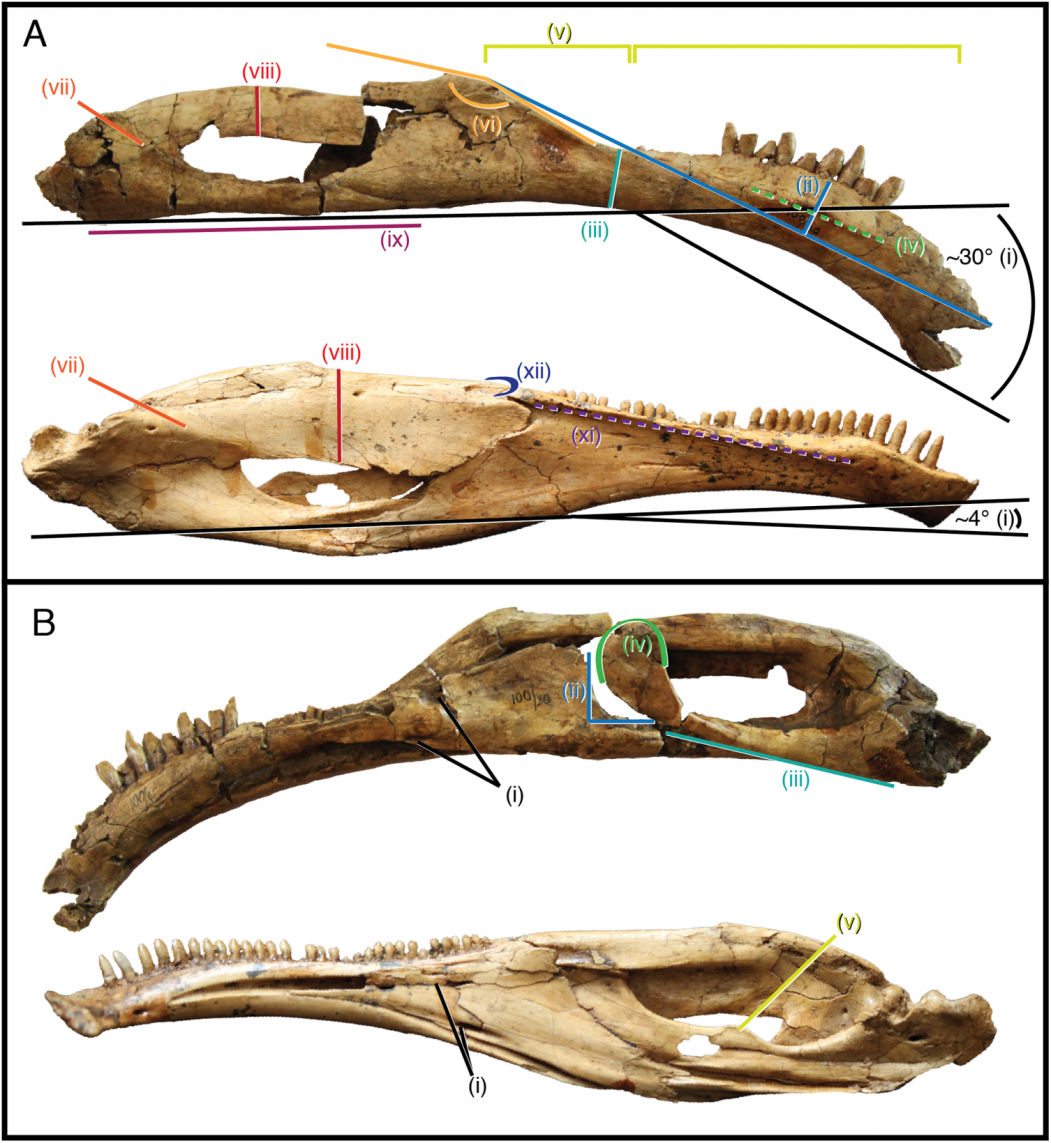
Differentially diagnostic features of *Segnosaurus galbinensis* MPC-D 100/80 and *Erlikosaurus andrewsi*. (A) Right hemimandible of *Segnosaurus galbinensis* (superior) and left hemimandible of *Erlikosaurus andrewsi* (inferior, reversed) in lateral view; (B) Right hemimandible of *Segnosaurus galbinensis* (superior) and left hemimandible of *Erlikosaurus andrewsi* (inferior, reversed) in medial view. Abbreviations (i–xii) refer to diagnostic traits discussed in text. Elements scaled to same size.

As it stands to date, the following features of *S. galbinensis* (Fig. 6) are otherwise unknown in Therizinosauria and are therefore differentially diagnostic: (a) ratio of edentulous rostral dentary length to tooth row length approximately 1:5; (b) pronounced ventral deflection of rostral dentary (∼30°) (Fig. 6Ai); (c) dentary tooth row arced, over half the dorsoventral height dorsal to transverse axis of dentigerous portion of dentary (Fig. 6Aii); (d) minimum dorsoventral height of dentary caudal to tooth row, dentary constricted between caudalmost tooth row and contact with surangular (Fig. 6Aiii); (e) lateral shelf located midway between dorsal and ventral margins of dentary (Fig. 6Aiv); (f) significant proportion (approx. 1/4th) of the dorsal margin of the dentary edentulous caudal to tooth row, tooth row does not extend to surangular articulation (Fig. 6Av); (g) medial margin of dental alveolus crenulate (scalloped); (h) dorsal margin of dentary/surangular articulation kinked, elements conjoin at 160° angle (Fig. 6Avi); (i) ventral foramen of splenial located rostral to dorsal foramen (Fig. 6Bi); (j) caudal margin of splenial bears a near 90° notch to receive prearticular (Fig. 6Bii); (k) lateral shelf on surangular bridging caudodorsal external mandibular foramen and articular absent (Fig. 6Avii); (l) lateral flange of surangular dorsoventrally shallow (Fig. 6Aviii); (m) ventral aspect of angular flat; prearticular ventrally displaced, caudal aspect contributes to ventral margin of hemimandible in medial view (Fig. 6Aix); (n) body of prearticular relatively straight (Fig. 6Biii); (o) rostralmost aspect of prearticular terminates in an expanded, subcircular tab (Fig. 6Biv); (p) mesial carina of tooth crown folded distally and serrated (Figs. 3D and 3E); (q) extracarinal denticles populate mesial face of tooth crown (Figs. 3B, 3C and 3F); (r) basal aspect of mesial carina bears serrated, triangular facet at cervix (Figs. 3G, 3H, 3J and 3K) (s) distalmost lateral dentary teeth subconidont, bearing triple carinae (Fig. 5).

Likewise, the following traits exhibited by *E. andrewsi* (Fig. 4) are otherwise unknown in Therizinosauria and may serve to differentially diagnose this taxon: (a) ratio of edentulous rostral dentary length to tooth row length approximately 1:8; (b) slight ventral deflection of the rostral dentary (∼4°) (Fig. 6Ai); (c) lateral shelf on dentary extensive, reaches caudal termination of tooth row (Fig. 6Axi); (d) lateral shelf sharp, distinctly raised from lateral aspect of dentary (Fig. 6Axi); (e) tooth row extends to surangular articulation (Fig. 6A); (f) caudodorsal process of dentary visible in lateral view (Fig. 6Axii); (g) ventral foramen of splenial located caudal to dorsal foramen (Fig. 6Bi); (h) lateral shelf on surangular bridging caudodorsal external mandibular foramen and articular; (Fig. 6Avii) lateral flange of surangular dorsoventrally deep (Fig. 6Aviii); (k) prearticular bears dorsal tab (Fig. 6Bv).

### Paleobiological considerations

The diet of therizinosaurian theropod dinosaurs is largely regarded to fall within the omnivory-herbivory spectrum (e.g., Paul, 1984; Russell & Russell, 1993; Barrett, 2005; Kirkland et al., 2005; Zanno et al., 2009). Previous studies have hypothesized a phylogenetic trend of intensifying herbivory in the clade based on a combination of traits analogous with those of extant herbivores such as concomitant broadening of the pelvic cavity, elongation of the neck; reduction in relative cranial size (Zanno et al., 2009; Zanno, 2010), reduction in craniocervical musculature (Smith et al., 2011; Smith, 2014), reduction in bite force (Lautenschlager, 2013), and quantitatively correlated ecomorphology including rostral tooth loss, development of a rhamphotheca on the premaxilla and dentary, and a downturned rostral dentary in specialized taxa (Zanno, 2010; Zanno & Makovicky, 2011). Nonetheless, to date, overall tooth morphology—a key dietary indicator—has been considered relatively simplistic in therizinosaurians compared to that of many other herbivorous dinosaur clades (Erickson et al., 2012; Erickson et al., 2015). In therizinosaurians generally, the dentition is characterized only by weak trends of increasing tooth symmetry (e.g., *E. andrewsi*, Fig. 4), enlargement of denticles (e.g., *S. galbinensis*; Clark, Maryánska & Barsbold, 2004), and pseudoheterodonty (more conidont mesial dentition; sensu Hendrickx, Mateus & Araújo, 2015) in some taxa (Zanno et al., 2009; Zanno, 2010; Zanno & Makovicky, 2011). Unique dental specializations have thus far been limited to the cupped, incisiform rostral teeth of *F. utahensis* (Kirkland et al., 2005; Zanno, 2010), and the purportedly labially convex lateral teeth of *J. yixianensis* (Pu et al., 2013).

*Segnosaurus galbinensis* provides novel evidence of dental complexity, including apomorphic features that add to our understanding of ecomorphological diversity in the dentition of therizinosaurians. The presence of extracarinal denticles on the mesial tooth crown coupled with a mesial carinal fold appressed to a wedge-shaped, denticulated facet on the distal crown is a specialization that likely indicates a higher degree of oral processing than observed in other therizinosaurians. Taken together, these apomorphic tooth traits create a roughened, shredding surface near the base of the crowns in *S. galbinensis* that is also otherwise unknown in Theropoda.

At least three different geologic formations are purported to preserve multiple sympatric species of therizinosaurians (Barsbold & Perle, 1980; Zhang et al., 2001; Xu et al., 2002; Sues & Averianov, 2016), suggesting that niche partitioning between contemporary species may have played a key role in the evolutionary success of this clade. Functional investigations of ungual morphology in Therizinosauria indicate that members of the clade exploited a diversity of foraging strategies during their evolution (Lautenschlager, 2014). However, to date, biomechanical studies have not been used to contrast food processing or foraging strategies in sympatric therizinosaurians. The contrast of highly specialized dentition in *S. galbinensis*, and relatively amorphous dentary crowns in the contemporary taxon *E. andrewsi* (Fig. 4) supports a hypothesis of dietary partitioning between sympatric therizinosaurians inhabiting Asian ecosystems during the Late Cretaceous, a conclusion strengthened by the extreme difference in body mass estimated for sympatric therizinosaurians from the Bayanshiree Formation (up to 500%; Zanno & Makovicky, 2012).

## Conclusions

*Segnosaurus galbinensis*, a large-bodied therizinosaurid from the Upper Cretaceous Bayanshiree Formation, Gobi Desert, Mongolia, possesses a number of apomorphic features of the mandible and dentition. These include gracile, linear postdentary elements that contribute to a subrectangular caudal hemimandible, approximately 30° of ventral deflection to the rostral dentary, edentulism of the caudal dentary, and a thickened lateral shelf, as well as a number of additional mandibular traits currently useful for differentiating *S. galbinensis* from other therizinosaurians. Apomorphic dental traits include triple carinae on the distalmost dentary teeth, lingually folded mesial carinae appressed against triangular denticulate facets on the distal carinae, and extracarinal accessory denticles. These traits, taken together with crown wear in the clade (Clark, Maryánska & Barsbold, 2004), are indicative of increased shredding capabilities and a higher degree of oral processing than hypothesized for other therizinosaurians.

The highly derived dental specializations of *S. galbinensis*, including features apomorphic for Theropoda, suggest this taxon persisted on unique food resources and/or employed novel feeding strategies that add to new data to existing hypotheses of facultative or obligate herbivory in therizinosaurians (e.g., Paul, 1984; Russell & Russel, 1993; Barrett, 2005; Kirkland et al., 2005; Zanno et al., 2009). The complex dental morphology of *S. galbinensis* contrasts with the simplistic dentition of the coeval Bayanshiree therizinosaurid *E. andrewsi* (Barsbold & Perle, 1980; Clark, Perle & Norell, 1994; Clark, Maryánska & Barsbold, 2004; Lautenschlager et al., 2012; Lautenschlager et al., 2014), providing strong support for niche partitioning among therizinosaurid species inhabiting Asian ecosystems during the Late Cretaceous.

## Additional Information and Declarations

### Data Deposition

The following information was supplied regarding data availability: All raw data generated by the research in this article is included with the publication.
